# Dynamics of NO interacting with soluble guanylate cyclase from 1 ps to 0.1 s and induced structural transitions

**DOI:** 10.1186/1471-2210-11-S1-P77

**Published:** 2011-08-01

**Authors:** Byung-Kuk Yoo, Isabelle Lamarre, Jean-Louis Martin, Fabrice Rappaport, Michel Negrerie

**Affiliations:** 1 Laboratoire d'Optique et Biosciences, INSERM, Ecole Polytechnique, Palaiseau, France; 2 Institut de Biologie Physico-Chimie, CNRS, Paris, France

## Results

We investigated the interaction between purified soluble guanylate cyclase (sGC) from beef lung and NO by time-resolved spectroscopy in a time-range which encompasses eleven orders of magnitude, from 1 ps [1] to 0.1 s [2]. After its dissociation from the heme, NO either recombines geminately to the 4-coordinate heme within τ_G1_ = 7 ps [1] (96 ± 1 % of the population) or exits the heme pocket (4 ± 1 %), allowing the proximal histidine to rebind within 62 ± 10 ps. Then, NO is distributed in two approximately equal populations (~2 %). One geminately rebinds to the 5-coordinate heme (τ_G2_ = 6.5 ns) while the other migrates into the solution. NO can rebind from the solution (bimolecular rebinding, τ_B_ = 0.25 ms with [NO] = 20 µM), forming a 6-coordinate heme with a rate constant of 2 x 10^8^ M^-1^ s^-1^ (*in vitro* purified protein) very close to that measured in platelets (3 × 10^8^ M^-1^s^-1^) [3].

The cleavage of Fe-His bond and subsequent formation of 5-coordinate NO-heme occurs with different time constants for NO which geminately rebinds (τ_5C1_ = 0.66 µs) and for NO which binds from the solution (τ_5C2_ = 43 ms). Thus, because the same structural event occurs with rates separated by more than 4 orders of magnitude, we must infer that sGC is not in the same structural state in both cases, with a different strain exerted on the Fe-His bond. This allosteric transition between both states occurs in the time range 0.66 µs < τ_R_ < 250 µs in sGC after His rebinding and NO release.

## Conclusion

Since the discovery that NO binds to the proximal heme side in cytochrome c' [4] (AXCP), several models of sGC activation were proposed which include the binding of NO to the proximal heme side despite the lack of observation for such an activation step in sGC. After the fast histidine rebinding in the picosecond range, we have observed only four phases in the nano to millisecond time range (assigned as indicated in Table 1). Thus, analysis of the entire NO dynamics from 1 ps to 0.1 s did not detect NO binding to the proximal side of sGC heme despite the fact that NO shows the same geminate rebinding to the 4-coordinate heme in sGC and AXCP [5]. Our data can be described with a one-site model, without phases assigned to dinitrosyl formation or to NO proximal binding.

**Table 1:** Rates of the transitions observed in kinetics.

*Transition*	*Time constants*	*Transition rates*
Bimolecular NO binding to 5c-His	τ_B_ = 0.25 ms; [NO]= 20 µM	*k*_B_ = 2 x 10^8^ M^-1^s^-1^
Conversion 6c-NO → 5c-NO	τ_5C2_ = 43 ms	*k_5C_*_2_ = 23 s^-1^
Geminate NO rebinding to 5c-His	τ_G2_ = 6.5 ns	*k*_G2_ = 0.15 x 10^9^ s^-1^
Conversion 6c*-NO → 5c*-NO	τ_5C1_ = 0.66 µs	*k*_5C1_ = 1.5 x 10^6^ s^-1^
His rebinding to 4c-heme	τ_His_ = 62 ps	*k*_His_ = 1.4 x 10^10^ s^-1^
Geminate NO rebinding to 4c-heme	τ_G1_ = 7 ps	*k*_G1_ = 0.13 x 10^12^ s^-1^
Structural relaxation sGC* → sGC	0.66 µs < τ_R_ < 250 µs	4 x 10^3^ s^-1^ <*k*_R_ <1.5 x 10^6^ s^-1^

**Figure 1:**
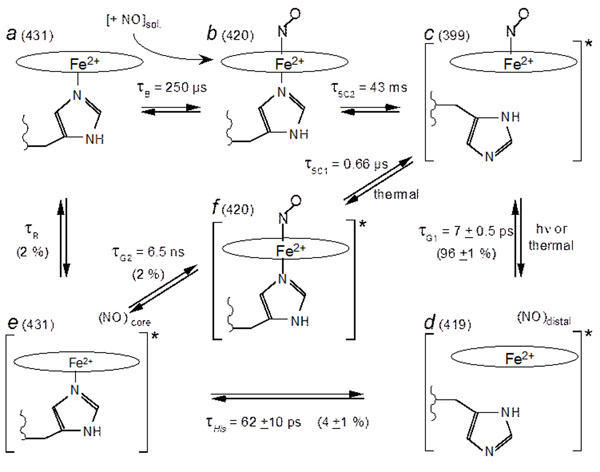
Model describing the species and transitions involved in the time-resolved experiments. ***a***: 5-c-His sGC in the resting state. ***b***: 6-C sGC. ***c***: 5-c-NO sGC, in the activate state. ***d***: 4-c sGC after NO dissociation. ***e***: 5-c-His sGC still in the activated conformation, immediately after His105 rebinding. ***f***: 6-c sGC in the activated state, immediately after NO geminate rebinding. The value adjacent to the letter label is the wavelength of the species. The star at the right bracket in *c*, *d*, *e* denotes the activated state of sGC. The starting species of the kinetic measurements is *c*. The time constants indicated are those measured and the corresponding rates are given in Table 1. In species *d*, NO is located within the heme pocket whereas in species *e*, NO is located in another docking site in the protein.
